# The effects of a non-cognitive versus cognitive admission procedure within cohorts in one medical school

**DOI:** 10.1007/s10459-017-9782-1

**Published:** 2017-06-10

**Authors:** Marieke de Visser, Cornelia Fluit, Janke Cohen-Schotanus, Roland Laan

**Affiliations:** 1 Department for Research in Learning and Education, Radboudumc Health Academy, Radboud University Medical Center, Postbus 9101, Huispost 43, 6500 HB Nijmegen, The Netherlands; 2 Institute for Medical Education, University Medical Center Groningen, University of Groningen, Groningen, The Netherlands

**Keywords:** Medical school, Academic performance, Cognitive selection, Non-cognitive selection

## Abstract

In medical school selection, non-cognitive performance in particular correlates with performance in clinical practice. It is arguable, therefore, that selection should focus on non-cognitive aspects despite the predictive value of prior cognitive performance for early medical school performance. The aim of this study at Radboud University Medical Center, the Netherlands, is to determine the effects of admitting students through an autonomous non-cognitive procedure on early medical school performance. We compared their performance to the performance of students selected through an autonomous cognitive selection procedure, enrolling in the Bachelor’s curriculum simultaneously. 574 students (2013 and 2014 cohorts), admitted through non-cognitive selection (based on portfolio, CASPer and MMI, n = 135) or cognitive selection (curriculum sample selection, n = 439) were included in the study. We compared dropout rates, course credits and grades, using logistic and linear regression. The dropout rate was the highest in the non-cognitive selection group (*p* < 0.001). Students admitted through non-cognitive selection more often obtained the highest grade for the nursing attachment (*p* = 0.02) and had a higher mean grade for the practical clinical course in year 3 (*p* = .04). No differences in course grades were found. The results indicate that students perform best on the elements of the curriculum that are represented most strongly in the selection procedure they had participated in. We recommend the use of curriculum sample procedures, resembling the early medical school curriculum,—whether it has a more cognitive or a more non-cognitive focus—, to select the students who are likely to be successful in the subsequent curriculum.

## Introduction

A wide range of procedures is currently in use in student selection for medical school, and assessment tools also vary widely in terms of their content, characteristics, and number. In a comprehensive systematic review, Patterson et al. (2016) have recently shown that academic records, Multiple Mini Interviews (MMIs), and Situational Judgment Tests (SJTs) are among the most effective selection methods. They observe that “achievement in different selection methods may differentially predict performance at the various stages of medical education and clinical practice” (Patterson et al. 2016). Prior cognitive performance (Grade Point Average, GPA) has shown to be a predictor of medical school performance in the early years of medical school (Ferguson et al. 2002; Siu and Reiter 2009). Non-cognitive performance, however, correlates with performance in clinical practice, in terms of scores on Objective Structured Clinical Examinations (OSCEs), assessment of professional behavior in clinical placements, clerkship evaluations, and clinical examination-based licensing examination scores (Adam et al. 2015; Eva et al. 2009; Reiter et al. 2007).

As professional performance in clinical practice is the key outcome of medical education, one might argue that student selection should focus on non-cognitive aspects. However, selection on non-cognitive aspects may have adverse side-effects on performance in early, mainly cognitive, medical school, which is best predicted by cognitive selection methods or academic records. Some studies have already correlated medical school performance with non-cognitive and cognitive elements of a preceding mixed selection procedure, for instance (Adam et al. 2015), or have compared the effects of non-cognitive versus cognitive selection in different cohorts or programs (Lucieer et al. 2015). Adam et al. described an autonomous contribution of both cognitive and non-cognitive elements within a mixed procedure to medical school performance. Lucieer et al., comparing a cognitively selected cohort and a non-cognitively selected cohort, have found no differences in year 1 GPA nor in the probabilities of passing the third-year OSCE or obtaining the Bachelor’s degree within 3 years.

The designs of the above-mentioned studies have important limitations, however. A main limitation of the study by Adams et al. is that it concerns a mixed procedure. All admitted students, therefore, showed sufficient performance on both aspects. The non-cognitive selection in the study by Lucieer et al., was based on a portfolio of extracurricular activities only, the predictive value of which is questionable (Patterson et al. 2016), and the study compares different cohorts. To be able to draw valid conclusions about the autonomous effects of non-cognitive selection on medical school performance, the non-cognitive procedure should be compared to a control group, and, in the design, each procedure should be carried out independently, and the applicable curriculum should be the same. We have not found any studies comparing the performance of students admitted through an autonomous non-cognitive versus an autonomous cognitive procedure *within* cohorts, enrolling in exactly the same medical school program simultaneously. Therefore, the validity of findings is limited as variables other than only the variable under study may partly explain the potential differences between groups.

It is important to notice, furthermore, that terms like non-cognitive and non-academic are used in different ways in the domain of medical education and are relatively inaccurate, an issue that has been addressed in the research agenda on the validity of non-academic assessments, as recently proposed by Kreiter (2016) in this journal. He uses the term non-academic to refer to “virtually all medical school admission assessments that do not specifically target academic or cognitive ability”. In the current study, the term “non-cognitive” has been used in line with Kreiter’s definition of “non-academic” and, more specifically, it has been used to refer to the communicator, collaborator, health advocate, and professional competencies of the CanMeds model (Frank et al. 2015). These competencies require interpersonal skills, empathy, ethical decision-making, and the capacity to reflect on one’s behavior and act accordingly. The term “cognitive” refers to the capacity to acquire, process, and utilize knowledge.

The aim of the current study is to determine the effects of admitting students through a non-cognitive procedure on early (mainly cognitive) medical school performance. To meet this aim, we compared the early medical school performance of students selected through an autonomous non-cognitive selection procedure to the performance of students selected through an autonomous cognitive selection procedure within cohorts in one medical school. We hypothesized that students admitted through a non-cognitive procedure would 1. outperform students admitted through a cognitive procedure in non-cognitive medical school performance, and would 2. underperform in cognitive medical school performance.

## Methods

### Setting

This study was performed at the Radboud University Medical Center in Nijmegen, the Netherlands (RUMC). Each year, 330 new students are admitted to the RUMC medical school.

The study of medicine involves a 6-year program that follows directly after graduation from (pre-university level) secondary school, mostly at the age of 18. A 3-year mainly theoretical Bachelor’s program is followed by a 3-year Master’s program with mainly clinical rotations.

The RUMC curriculum consists of ten four-week courses in the first as well as the second year, each followed by a summative exam. This system continues in the first part of the third year; in the last part of the third year, students choose from a range of courses during five four-week periods. Though the Bachelor’s curriculum has a theoretical focus, two Bachelor’s courses focus on practical training. In the first year, nursing attachment students work in a nursery home or hospital ward (Helmich et al. 2012). They are assessed in a summary report of their supervisor, with input from multiple professionals who worked with the student. In the third-year practical clinical course, they are introduced to history taking, physical examination, and clinical reasoning and their competencies are assessed by six independent assessors in an assessment center. Medical doctors as well as simulated patients are involved in practical assignments like physical examinations, and some written assignments (on interpretation of cardiac sounds, for instance) are assessed by teachers.

### Population

In September 2013 and September 2014, a total number of 660 students enrolled in their Bachelor’s program in medical education at the RUMC. Out of these 660 students, 86 were excluded from the present study because: (a) they had enrolled in an individual track after relevant prior education at a university level (n = 5); (b) they had been admitted on grounds of their high pu-GPA [by law, students in the Netherlands have direct access to medical school if their pu-GPA is equal to or higher than 8 on a scale of 1 (poor)–10 (excellent)], (n = 76); or (c) they had taken fewer than two course exams in year one (n = 5). A total of 574 students were included.

### Selection procedures

To meet the aim of our study, we set up a design applying two independent selection procedures within each of the two cohorts: non-cognitive selection and cognitive selection.

#### Non-cognitive selection procedure

This procedure was open to all applicants who were about to finish secondary school, as well as to applicants with a different educational background meeting the conditions for admission to Dutch medical education (Ten Cate 2007). This procedure included three consecutive rounds. Participants could be rejected after each round. The procedure is considered mainly non-cognitive, as the focus was on the CanMeds roles of communicator, collaborator, health advocate, and professional. In the first round, as described below, part of the students qualified for the second round through cognitive performance. In the first round, applicants were asked to send in a portfolio. The portfolio consisted of three parts. In the first part, applicants had to prove that they had (A) a GPA of at least 6.75 (scale 1–10) in their pre-final year of secondary school (applicable to those finishing pre-university education at the time of selection) or were ranked in the top quartile of their cohort (applicable to applicants not finishing secondary school at the time of selection), or (B) substantial experience in a healthcare context as a volunteer or side job, or (C) substantial experience on an organizing committee or student board, or excellent performance in science, literature, arts, or sports. In the second part, applicants had to write a motivation letter explaining why they wanted to go to medical school and why they had specifically chosen the RUMC. In the third part, they had to send in three reference letters and their own reflection on these letters.

Each portfolio was independently assessed by two assessors. In this first round, the first part was assessed on content, and the second and third parts were just checked for their presence. If the content of the first part did not meet the above-mentioned criteria A, B or C and/or if the other parts proved to be incomplete, applicants were rejected. If the two assessors disagreed with each other, a third assessor was asked for discussion until consensus was reached.

In the second round, applicants sat an on-site computer-based exam. The exam was based on the principles of the descriptive questions part of CASPer (Dore et al. 2009), developed by McMaster University. Five short videos (each between 1 and 3 min) were shown, and applicants were asked to answer open-ended questions (20 in total) based on these videos. The situations shown in the videos related to the CanMeds competencies of communicator, collaborator, leader, health advocate, scholar, and professional (Frank et al. 2015). The answers were assessed by just one assessor per question, based on aspects of a correct answer that had been defined beforehand. This assessor was either a medical doctor or a senior medical student trained for this task by a medical doctor, who was available for reactive supervision.

An example of a video presented to the applicants is a 2-min fragment of patient–doctor communication in an emergency department, while the patient’s medical issue does not seem to be very urgent. The question asked was: “What elements in the doctor’s non-verbal communication have you seen that are appropriate to this situation?” Aspects in the answer that were rewarded with a credit were: she makes eye-contact with the patient; she sits (as opposed to stands) next to him; and she turns her body towards him.

For practical reasons, a maximum of the 90 best-scoring applicants were admitted to the third round, in which the applicants did an onsite assessment, being presented with several simulated situations in which they were supposed to act. This round was designed using the principles of MMIs (Eva et al. 2004; Rees et al. 2016). Applicants were assessed in situations evoking the use of the CanMeds competencies of communicator, health advocate, and professional; assessment was based on a 1–10 scale on predefined criteria and performed by an assessor or a trained actor involved in the scenario, depending on the scenario.

In addition, each applicant had two interviews: one relating to his/her motivation letter and one relating to his/her reference letters and supplemental personal reflection. Both interviews were assessed on reflection criteria by the interviewer. Each situation/interview was assessed by an independent assessor, so per applicant multiple independent assessors were involved.

All actors were experienced in the field of medical education and had been trained twice for the specific scenario, using a student actor. All interviewers and assessors were medical doctors who were teachers in the Bachelor’s curriculum. In the final score of this procedure, the score of the second round and the average score on the elements of the third round both counted for 50%, and a final ranking was made.

#### Cognitive selection procedure

Our cognitive selection procedure was open to all applicants who were about to finish secondary school. This procedure can be characterized as curriculum sample selection, as described in detail in a previous study (de Visser et al. 2017). Once admitted to the selection procedure, applicants were enrolled in a course in Blackboard, the digital learning platform used by the RUMC. The course topic was Diabetes. Basic biomedical, clinical, sociological, ethical, and psychological perspectives were integrated into the course, like in the medical school curriculum. Applicants took the online course at home during 4 weeks, and the estimated course load was 80 h. The course comprised lectures, assignments, and forums, simulating real medical education in Nijmegen. The forums allowed applicants to discuss topics related to the course and to ask and answer questions. Teachers moderated the forums to some extent and corrected apparent misconceptions. The procedure was considered to be mainly cognitive as applicants predominantly had to acquire, process, and utilize knowledge to be successful.

After their preparation period, applicants took an on-site multiple choice test (70% weight in final score) and wrote an essay focusing on psychological, ethical, and social aspects of the study subject (30%). Besides content aspects, essays were also assessed on structure, language, and writing style. The test was taken by 392 applicants in 2013 and 454 in 2014. Applicants rejected after curriculum sample selection were allowed to participate in the non-cognitive selection procedure the year after.

Applicants’ scores in the non-cognitive selection and the curriculum sample selection were ranked in one final ranking, alternating applicants from both procedures in the ratio of the number of applicants originally entering each procedure. After final scores had been ranked, 199 (2013) and 240 (2014) applicants were admitted through curriculum sample selection and 74 (2013) and 61 (2014) through non-cognitive selection. All other available places were taken by students who had direct access through high pu-GPA.

### Measures

The primary outcome measure was the dropout rate. In line with the RUMC academic dismissal policy, students obtaining fewer than 42 out of 60 credits in year 1 have to leave medical school by the end of year 1.

Additional outcome measures were grades for theoretical as well as practical performance. For these grades, we included a student’s first examination attempt only.

For cognitive performance, we defined the variables:

1. Average grade point for theoretical exams in year 1 [scale: 1 (poor)–10 (excellent)], excluding dropouts (n = 18);
2. Average grade point in second- and third-year theoretical exams.

For non-cognitive performance, we defined the variables:

3. Percentage of students receiving the maximum grade for the first-year nursing attachment (scale: insufficient- good–excellent);
4. Average grade point for practical clinical course in year 3.

As the 2013 cohort was the only one to finish the Bachelor’s program within the timeframe of our study, outcome measures 2 and 4 are applicable to this cohort only. All other outcome measures apply to both cohorts.

Post hoc, in a case control design, we compared the characteristics (age, sex, pu-GPA, and participation in the curriculum sample procedure the year before or not) of the non-cognitive selection dropouts to the non-cognitive selection admissions who did not drop out.

### Data collection

Pu-GPA data were made available by the Ministry of Education for this research. All other data were collected from the RUMC student and admission administrations. All data were treated as strictly confidential and were available for the researchers only, in conformity with our Medical Center’s privacy regulations. All analyses were conducted anonymously, and no results can be traced back to individual students. By Dutch law, no ethical approval is applicable to studies like ours, using regularly registered data anonymously. Therefore, our institute waived approval.

### Data analysis

For the descriptive data except age and for all outcome measures, we tested whether the two admission categories differed, using χ^2^ tests (categorical variables) or *t* tests (continuous variables). For age, a Mann–Whitney test was done, as testing showed that the distribution of the data was non-normal for this variable.

If significant differences were found on the outcome measures, we controlled for confounders through logistic or linear regression, as applicable. To control for secondary school performance, we adjusted for pu-GPA. This was computed as a mean score on the five subjects that all students had in common. Additionally, we controlled for sex and age if their addition to the regression model influenced the effect (regression coefficients) of the independent variable “admission route” for more than 10% (Grobbee and Hoes 2009). In the design of the study, the hypotheses we had about the direction of the effects, based on the literature, led us to calculate one-sided *p* values. Finally, we calculated Cohen’s d for effects found, to evaluate the effect sizes of continuous variables. The Statistical Package for the Social Sciences (SPSS) Windows version 20 was used for the statistical analyses.

## Results

### Descriptives

Descriptive statistics of students per admission route are shown in Table 1. No differences in the percentage of female students were found. Non-cognitive selection students had a higher median age and a lower pu-GPA than curriculum sample selection students.

**Table 1:** Descriptives

	Cognitive selection (1)	Non-cogn. selection (2)	Total
n	439	135	574
% female	65.1	67.4	65.7
Median age (years)*	18.4	19.8	18.6
Pu-GPA (SD)**	7.0 (.46)	6.8 (.52)	7.0 (.48)

Pu-GPA is composed of the grade points for the five subjects all students had in common: Dutch language, English language, biology, physics, and chemistry

* 1 < 2, *p* < 0.001; ** 1 > 2, *p* = 0.01

Of the students admitted through non-cognitive selection, 67 qualified for the second round of their procedure through option A (GPA), 20 through B (healthcare), and 48 through C (substantial experience on a student board or excellent performance in arts or sports). Of the students admitted through non-cognitive selection, 17 (12%) had participated in the cognitive procedure the year before.

### Dropout rate

Students admitted through non-cognitive selection showed a higher dropout rate in year one than students admitted through curriculum sample selection (χ_(1)_^2^ = 18.38, *p* = 0.00; Table 2). Adjusted for pu-GPA and age, the difference remains significant (*p* = .02; Table 3).

**Table 2:** Results of students of two admission routes

	Cognitive selection		Non-cognitive selection		Total
		N		N	
Dropout percentage*	1.6	439	8.1	135	3.1
Average grade point in year 1 theoretical exams (SD)	6.9 (.72)	433	6.9 (.78)	124	6.9 (.73)
Average grade point in years 2–3 theoretical exams (SD)	6.9 (.75)	193	6.9 (.81)	70	6.9 (.76)
Maximum grade for the nursing attachment*	75.9	439	85.2	135	77.9
Average grade point in year 3 practical clinical course (SD)*^#^	6.8 (.68)	176	7.0 (.59)	62	6.8 (.76)

* *p* < 0.05

^#^Cohen’s d: 0.3

**Table 3:** Dropout, nursing attachment maximum grade, and practical clinical course mean grade, adjusted for confounders

	N	% of students	β	*p*	OR
Dropouts*					
Cognitive selection	438	1.6	Ref	Ref	Ref
Non-cognitive selection	118	8.5	1.26	.02	3.5
Nursing attachment maximum grade**					
Cognitive selection	437	75.7	Ref	Ref	Ref
Non-cognitive selection	109	87.2	.71	.02	2.0

Practical clinical course*	N	Mean grade	β	t	*p*
Cognitive selection	175	6.8	Ref	Ref	Ref
Non-cognitive selection	56	7.0	.20	1.78	.04

(N is lower than in Table 2 because of pu-GPA missing data)

* Adjusted for pu-GPA and age, which were the only confounders. Significant difference at a *p* ≤ 0.05 level

** Adjusted for pu-GPA, which was the only confounder. Significant difference at a *p* ≤ 0.05 level

### Average grade point for theoretical exams in year 1

No significant difference between the groups was found concerning the average grade point on theoretical exams in year one (t = 1.21, *p* = 0.42, CI −1.3;.16; Table 2).

### Average grade point in 2nd and 3rd year theoretical exams

No significant difference between the groups was found concerning the average grade point on theoretical exams in years two and three (t = .03, *p* = .41, CI −.21;.21; Table 2).

### Percentage of students receiving the maximum grade for the first-year nursing attachment

Students admitted through non-cognitive selection obtained the highest grade for the nursing attachment more often than students admitted through curriculum sample selection (χ_(1)_^2^ = 4.89, *p* = 0.01; Table 2). Adjusted for pu-GPA, the difference remains significant (*p* = .02; Table 3).

### Average grade point for practical clinical course in year 3

Students admitted through non-cognitive selection had a higher average grade point for the practical clinical course in year 3 than students admitted through cognitive selection (t = −1.85, *p* = .03, CI −.37;.12). Adjusted for pu-GPA and age, the difference remains significant (*p* = .04; Table 3).

### Non-cognitive selection: dropouts versus non-dropouts

Within the non-cognitive selection group, dropouts had a lower pu-GPA than non-dropouts [6.4 (SD .49) vs 6.9 (SD .50)] (t = 3.10, *p* = .002, CI .18;.84). Of the dropouts, 60% had a pu-GPA ≤ 6.5 compared to 26% of the non-dropouts. No differences were found between the groups in mean age, sex, and whether or not they had been rejected after participation in the curriculum sample procedure before (detailed data not shown).

## Discussion

The aim of this study was to determine the effects of a non-cognitive admission procedure on *early* (i.e. mainly cognitive) medical school performance. To do so, we focused on differences between students who had been admitted through a cognitive selection procedure (curriculum sample selection) and students who had been admitted through a non-cognitive selection procedure.

Two main effects appear: (1) the dropout rate is the highest in the non-cognitive selection group, and (2) in non-cognitive performance (nursing attachment and practical clinical course), the students admitted through non-cognitive selection outperform students admitted through cognitive selection. Both effects confirm the hypotheses.

In the introduction section of this paper, we suggested that non-cognitive selection is preferable as it correlates to performance in clinical practice, which is the key outcome of medical education. The first effect of our study would appear to be worrisome in this respect: if students, despite their potential capacities for successful clinical practice, drop out in year 1, they will never even reach the clinical phase of their education. On the other hand, the merit of the non-cognitive selection procedure is that students most often obtain the maximum score for the nursing attachment and have a higher mean grade for the practical clinical course. This may be an indicator for better future clinical performance as research has shown that early non-cognitive performance predicts performance in clinical settings in terms of scores on Objective Structured Clinical Examinations (OSCEs), assessment of professional behavior in clinical placements, clerkship evaluations, and clinical examination-based licensing examination scores (Adam et al. 2015; Eva et al. 2009; Reiter et al. 2007). The effect size for the difference in grades for the practical clinical course is small (Kaasenbrood et al. 2010). Conclusions should be drawn with caution, as the influence of this significant difference on daily practice is unclear.

Despite the second effect, we are not concerned that the cognitive procedure will favor selection of “bookworms” (Eva et al. 2009), as around 76% of this group of students still obtain the maximum score (excellent) for their nursing attachment, and their mean grades for the practical clinical course are just slightly (albeit significantly) lower than those of the non-cognitive selection group.

Lastly, for the students who continue after year 1, no differences were found on average grade point on theoretical exams in year 1 and year 2 plus 3. As the dropout rate in year one is a direct result of lower grades, the difference in dropout rates may have masked a potential difference in grades in year 2 plus 3 due to attrition bias (Schmidt et al. 2012).

In the design of the non-cognitive selection procedure, one option for the applicants to qualify for the second round was a fairly cognitive one: a pu-GPA threshold or pu-performance in the top quartile of their cohort. For half the applicants in the non-cognitive procedure, therefore, the procedure was not entirely non-cognitive. This should be taken into account while interpreting the results. However, this cognitive performance only led to admission to the following phase of the procedure, consisting of two non-cognitive rounds, while the students’ performance in these rounds could not be counterbalanced through the cognitive qualification in the first round.

We also asked students to submit reference letters and a motivation statement in the first round although research shows these have limited predictive value for medical school performance (Patterson et al. 2016) and barely distinguish between applicants (Wouters et al. 2014). Therefore, we did not assess their content in the first round. Applicants were asked to submit these letters in the first round as their willingness to make an effort may be more predictive of future performance than success in the selection procedure as such and, thus, appears to be a mode of self-selection (O’Neill et al. 2011; Schripsema et al. 2014). The reference letters and the motivation statement were used at a later point in the MMI setting in the third round to reduce weaknesses inherent in non-structured interviews on this topic (Patterson et al. 2016).

Our findings are partly in line with the conclusions of the study by Lucieer et al. (2015), referred to in the Introduction section of this study. Our study adds to this by comparing an autonomous non-cognitive and an autonomous cognitive procedure *within* each of the two cohorts, and whereas Lucieer et al. based non-cognitive selection on a portfolio only, we added a CASPer-based and an MMI-based round, both of which have a stronger theoretical underpinning in predicting future performance. Like Lucieer et al., we did not find any differences between the groups in year 1 GPA, and our study shows that this pattern continues in years 2 and 3. Unlike Lucieer et al., who found no differences in third-year OSCE, we have found differences in the third-year clinical course. Lastly, Lucieer et al. did not find any differences in percentages of dropouts between cognitive and non-cognitive selection groups, which differs from our results as well. Their final conclusion that non-cognitive selection “is not sufficient to select the best academically performing students” can be confirmed by our results with respect to dropout rates.

To gain a better understanding of the above-mentioned statement in relation to our results, we compared the characteristics of students admitted through non-cognitive selection who dropped out to their counterparts who continued after year 1, although the subgroups were small. The dropouts have a lower pu-GPA compared to their counterparts. This indicates that adding a certain pu-GPA threshold (6.5) to the procedure would result in a lower dropout rate in this group. Nevertheless, doing so would reject 26% of successful students admitted through non-cognitive selection as well, which would be an inappropriate side-effect.

### Possible explanations

Research shows that the predictive value of selection methods may depend on the curriculum of the medical school (Edwards et al. 2013). At the time of our study, the Bachelor’s curriculum at the RUMC was mainly theoretical. In the current study, the cognitive procedure was a close sample of the curriculum, but the non-cognitive procedure was less so. Therefore, we think that the differences in dropout percentages between the groups may reflect the predictive value of curriculum sample selection as a method, instead of the predictive value of cognitive selection itself (de Visser et al. 2017; Prideaux et al. 2011).

Furthermore, students admitted through the cognitive selection procedure had all just left secondary school. Students admitted through the non-cognitive selection procedure had a wider variety of educational or professional backgrounds, and their comparative distance from study as a discipline at the time of entering medical school may explain differences in cognitive performance. For dropout rates this is the case, but no differences in theoretical course grades were found.

Besides evidence referred to in the introduction of this study, the fact that students admitted through non-cognitive selection outperform those admitted through cognitive selection in practical courses may be explained by a more practical attitude or an application-oriented learning style (Vermunt 1998). They may have the capacity to transform theory into practice more easily. Their performance is partly explained by age (confounder in the results of the practical clinical course). Particularly in the non-cognitive selection group, age may also represent years of experience in practical situations. After adjustment for age, however, the difference in performance remains significant. It is unclear whether this indicates that age did not represent experience (properly) or that experience plays a minor role in explaining clinical performance.

To further explore the influence of experience, a more exact measure of experience would be needed. Moreover, it would be worth comparing first-year clerkship results to gain more insight into whether the difference in performance in practical settings can be explained by years of experience.

### Strengths and limitations

The strength of this study is that it combines consecutive cohorts and is, to our knowledge, the first study comparing an autonomous non-cognitive and autonomous cognitive selection procedure within cohorts in one medical school. Some studies conclude that non-cognitive and cognitive performance correlate (Eva et al. 2009) and that non-academic skills and academic measures are not independent (Niessen and Meijer 2016). The current study is the first to disentangle the effects of both in isolation within cohorts in one medical school.

The study is limited by the fact that the follow-up is short. Because the students’ performance in clerkships could not be measured within the timeframe of the study, only a few non-cognitive outcome measures were available. Therefore, we cannot be sure whether the pattern found will continue after the Bachelor’s program in the practical Master’s. Another limitation is that the options for choosing their preferred selection procedure differed among applicants, as school leavers had the possibility to choose between the cognitive and the non-cognitive procedure, and non school leavers had to participate in the non-cognitive procedure. This has influenced the composition of the groups and has reduced the mechanism of self-selection for the non school leavers. Within this group, those who felt more competent in the cognitive procedure had to participate in the non-cognitive procedure and may have been rejected, although they could have been successful in the cognitive procedure. The school leavers will have considered their chances of being successful in either procedure and will have chosen strategically, based on required competencies and/or time available. This may have positively influenced their success rate in either procedure as a result of adequate self-selection.

### Implications for practice

If one had to choose one procedure from the current study, both procedures should be judged in terms of gains and losses. The gains of the cognitive procedure in our study are that the procedure is less labor-intensive and that dropout rates are lower, both of which contribute to lower costs. Moreover, there is a probability that the students’ Bachelor grades would be higher. The loss to be accepted, however, would be slightly lower clinical grades. Coaching students in non-cognitive aspects may possibly help to counterbalance this small loss in terms of effect size. The gain of the non-cognitive procedure, on the other hand, would be student performance in undergraduate clinical situations, which is an indicator for performance in clinical practice. The loss would be at higher dropout rate. In contexts allowing nationwide comparison of pre-university grades like ours, this could be counterbalanced by a moderate cognitive threshold for all applicants in a non-cognitive focused selection procedure.

Following the line of thought of Eva’s “chain of evidence” (Eva et al. 2009), one would expect that each phase of medical education makes its own contribution to predicting a successful career in medical education: Prior cognitive performance predicts early medical school performance, which in itself predicts medical school performance in the clinical phase, which in itself predicts performance in professional practice. The results of the current study indicate that students perform best on the elements of the early curriculum that are represented most strongly in the selection procedure they had participated in. Consequently, an important consideration for practice is to use curriculum sample procedures to admit the students who will perform best in the subsequent curriculum.

## Conclusion

In summary, the effect of non-cognitive selection compared to cognitive selection is a higher dropout rate and slightly better performance in non-cognitive courses. In the current study, the cognitive procedure was a close sample of the curriculum, but the non-cognitive procedure was less so. Based on the results found, we recommend the use of curriculum sample procedures. Selection assessments should resemble the early medical school curriculum—whether it has a more cognitive or a more non-cognitive focus—as closely as possible to select those students who are likely to be successful in the early curriculum, and, subsequently, in the next phases of medical school.
